# Aminoglycoside-Induced Hair Cell Death of Inner Ear Organs Causes Functional Deficits in Adult Zebrafish (*Danio rerio*)

**DOI:** 10.1371/journal.pone.0058755

**Published:** 2013-03-22

**Authors:** Phillip M. Uribe, Huifang Sun, Kevin Wang, James D. Asuncion, Qi Wang, Chien-Wei Chen, Peter S. Steyger, Michael E. Smith, Jonathan I. Matsui

**Affiliations:** 1 Department of Neuroscience, Pomona College, Claremont, California, United States of America; 2 Department of Biology and Biotechnology Center, Western Kentucky University, Bowling Green, Kentucky, United States of America; 3 Oregon Hearing Research Center, Department of Otolaryngology, Oregon Health & Science University, Portland, Oregon, United States of America; Texas A&M University, United States of America

## Abstract

Aminoglycoside antibiotics, like gentamicin, kill inner ear sensory hair cells in a variety of species including chickens, mice, and humans. The zebrafish (*Danio rerio*) has been used to study hair cell cytotoxicity in the lateral line organs of larval and adult animals. Little is known about whether aminoglycosides kill the hair cells within the inner ear of adult zebrafish. We report here the ototoxic effects of gentamicin on hair cells in the saccule, the putative hearing organ, and utricle of zebrafish. First, adult zebrafish received a single 30 mg/kg intraperitoneal injection of fluorescently-tagged gentamicin (GTTR) to determine the distribution of gentamicin within inner ear sensory epithelia. After 4 hours, GTTR was observed in hair cells throughout the saccular and utriclar sensory epithelia. To assess the ototoxic effects of gentamicin, adult zebrafish received a single 250 mg/kg *intraperitoneal* injection of gentamicin and, 24 hours later, auditory evoked potential recordings (AEPs) revealed significant shifts in auditory thresholds compared to untreated controls. Zebrafish were then euthanized, the inner ear fixed, and labeled for apoptotic cells (TUNEL reaction), and the stereociliary bundles of hair cells labeled with fluorescently-tagged phalloidin. Whole mounts of the saccule and utricle were imaged and cells counted. There were significantly more TUNEL-labeled cells found in both organs 4 hours after gentamicin injection compared to vehicle-injected controls. As expected, significantly fewer hair cell bundles were found along the rostral-caudal axis of the saccule and in the extrastriolar and striolar regions of the utricle in gentamicin-treated animals compared to untreated controls. Therefore, as in other species, gentamicin causes significant inner ear sensory hair cell death and auditory dysfunction in zebrafish.

## Introduction

Sensory hair cells are mechanoreceptors within the inner ear that transduce sound and head movements into neural signals making them essential for hearing and balance [1], [2], [3]. Loss of these receptors from excessive noise, ototoxic pharmaceutical agents, and aging can lead to permanent hearing loss and vestibular deficits in humans. Aminoglycoside antibiotics, like gentamicin, are a class of commonly-prescribed and essential antibiotics used to treat severe gram-negative bacterial infections, but they can cause permanent loss of sensory hair cells in humans and other mammals [4].

Zebrafish (*Danio rerio*) have become an established animal model to study sensory hair cell death [5], [6], [7], [8], [9], [10], [11], [12], [13], [14], [15], [16], [17], [18], [19], [20]. While zebrafish do not have a dedicated auditory organ like the mammalian cochlea, they have otolithic vestibular organs such as the saccule and utricle that are similar to mammalian vestibular organs. The zebrafish saccule, as in other teleosts, is thought to process auditory information [21], [22]. Zebrafish can detect auditory frequencies between 100 Hz and 4000 Hz [23], [24], and also have a lateral line system that detects changes in water flow and is composed of neuromasts along the fish’s head and body [7], [25], [26]. Each neuromast contains sensory hair cells and non-sensory supporting cells that project into the aquatic environment making it very convenient to study the effects of drugs on sensory hair cells *in vivo*.

The vast majority of sensory hair cell death studies in zebrafish have focused on lateral line neuromasts of larvae [6], [7], [8], [9], [10], [11], [12], [13], [14], [15], [16], [17], [18], [19], [20]. One recent study investigated the regeneration of lateral line hair cells in adult zebrafish [26] and other studies investigated the effect of loud sound on supporting cell proliferation in the saccule of adult zebrafish [27], [28], [29]. The effect of aminoglycosides on the sensory hair cells within the inner ear of zebrafish has yet to be reported.

The purpose of this study was to establish the extent of hair cell death in the zebrafish inner ear, specifically in the saccule and utricle, following systemic aminoglycoside administration. Our goal was to reliably cause a lesion and assess the extent of the resulting hearing loss by measuring auditory evoked potentials (AEPs). This type of recording is routinely used in humans to assess hearing performance and has been successfully applied in zebrafish [23], [24]. We found that gentamicin induced the loss of sensory hair cells across the entire saccule and utricle of adult zebrafish and this was accompanied by shifts in auditory thresholds.

## Materials and Methods

### Experimental Animals

Transgenic TG (Brn3c:GAP43-GFP)^s356t^ fish on the TL background (AKA Brn3c-GFP zebrafish (*Danio rerio*), a gift from Dr. Herwig Baier, University of California, San Francisco) [20], [30], [31], or wildtype zebrafish obtained from commercial suppliers were used for these experiments. Zebrafish were maintained on a 14 hour light/10 hour dark cycle using standard procedures [32]. This study was designed based on the recommendations in the Guide for the Care and Use of Laboratory Animals issued by the National Institutes of Health. The Institutional Animal Care and Use Committees of Western Kentucky University (Animal Welfare Assurance #A3558-01) and Pomona College (Animal Welfare Assurance #A3605-01) approved these experiments.

### Drug Administration

Adult Brn3c-GFP transgenic zebrafish were anaesthetized with MS-222 (tricaine methanosulfonate; Sigma-Aldrich Corporation, St. Louis, MO), weighed, and given either a single intraperitoneal injection of 30 mg/kg Texas-Red conjugated gentamicin (GTTR; [33]), 250 mg/kg gentamicin sulfate (Sigma), 30 mg/kg unconjugated Texas Red (Life Technologies, Carlsbad, CA), or phosphate-buffered saline (PBS; Sigma) using a micro-injector (World Precision Instruments, Sarasota, FL) [34]. The fish were placed into individual tanks and allowed to recover in the fish facility for 4 or 24 hours before being euthanized, decapitated, and the heads fixed in 4% paraformaldehyde (Sigma) overnight at 4°C. The heads were rinsed three times in PBS for ten minutes each. Saccules and utricles were isolated using microsurgical tools (Fine Science Tools, Foster City, CA) under a Nikon or Leica dissecting microscope [35].

**Figure 1:**
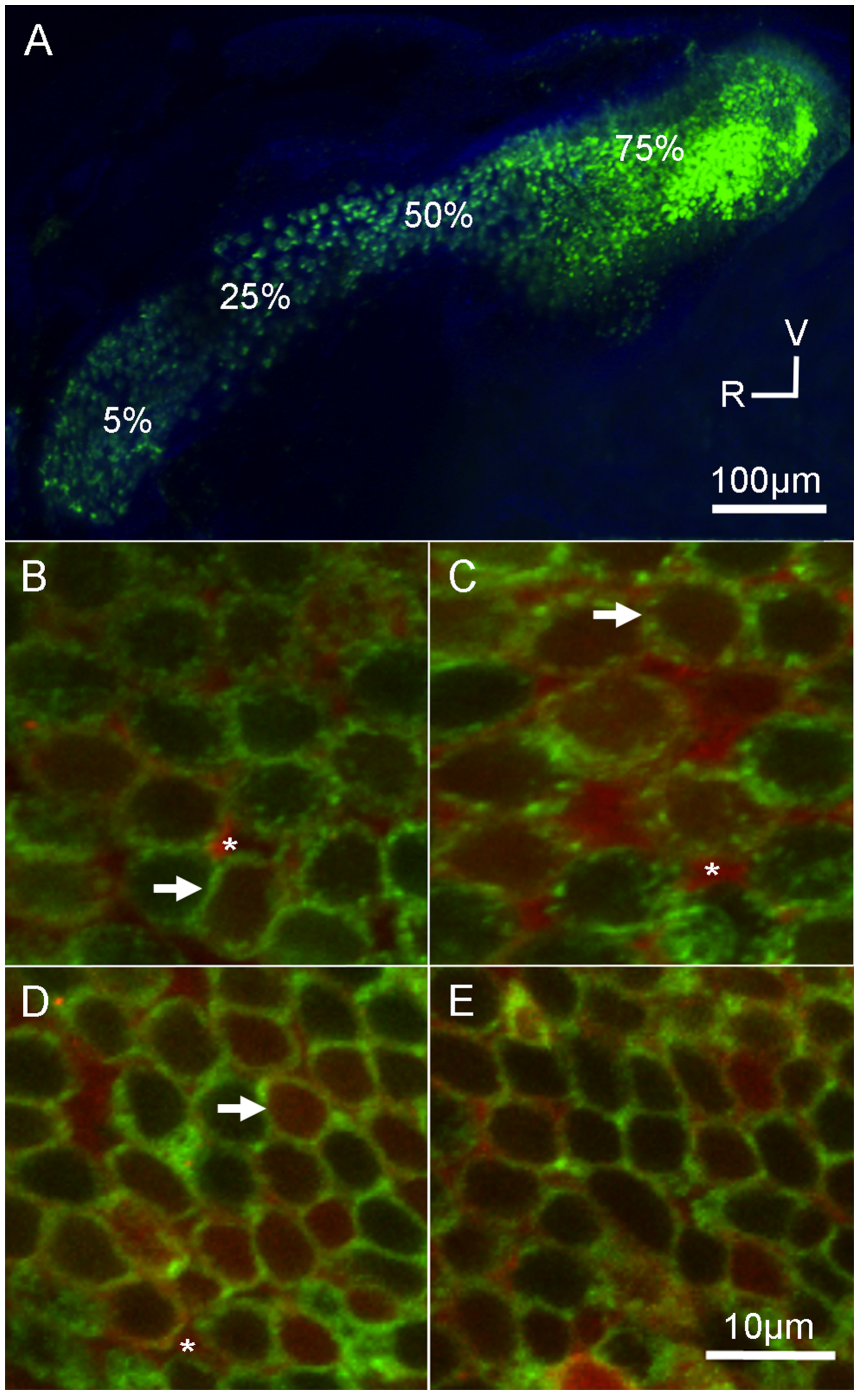
Gentamicin localizes to saccular hair cells. Adult Brn3c-GFP zebrafish received an intraperitoneal injection of Texas Red-conjugated gentamicin (GTTR; 30 mg/kg) and allowed to recover for 4 hours. (A) Low magnification image of the saccule. GFP-labeled hair cells (green) are located along the length of the organ. V = ventral, R = rostral. High magnification images (B–E) are single optical slices from individual *z*-stacks taken at (B) 5%, (C) 25%, (D) 50%, and (E) 75% along the length of the saccule from the rostral end (see A). GTTR fluorescence (red) was localized to hair cells (D, white arrows) and supporting cells (white asterisks) throughout the sensory epithelium. GFP fluorescence (green) is localized to the cell membrane region of hair cells. The *z*-stack slices are taken mid-way into the cell to show the localization patterns, thus neither hair cell nuclei nor stereociliary bundles are visible.

**Figure 2:**
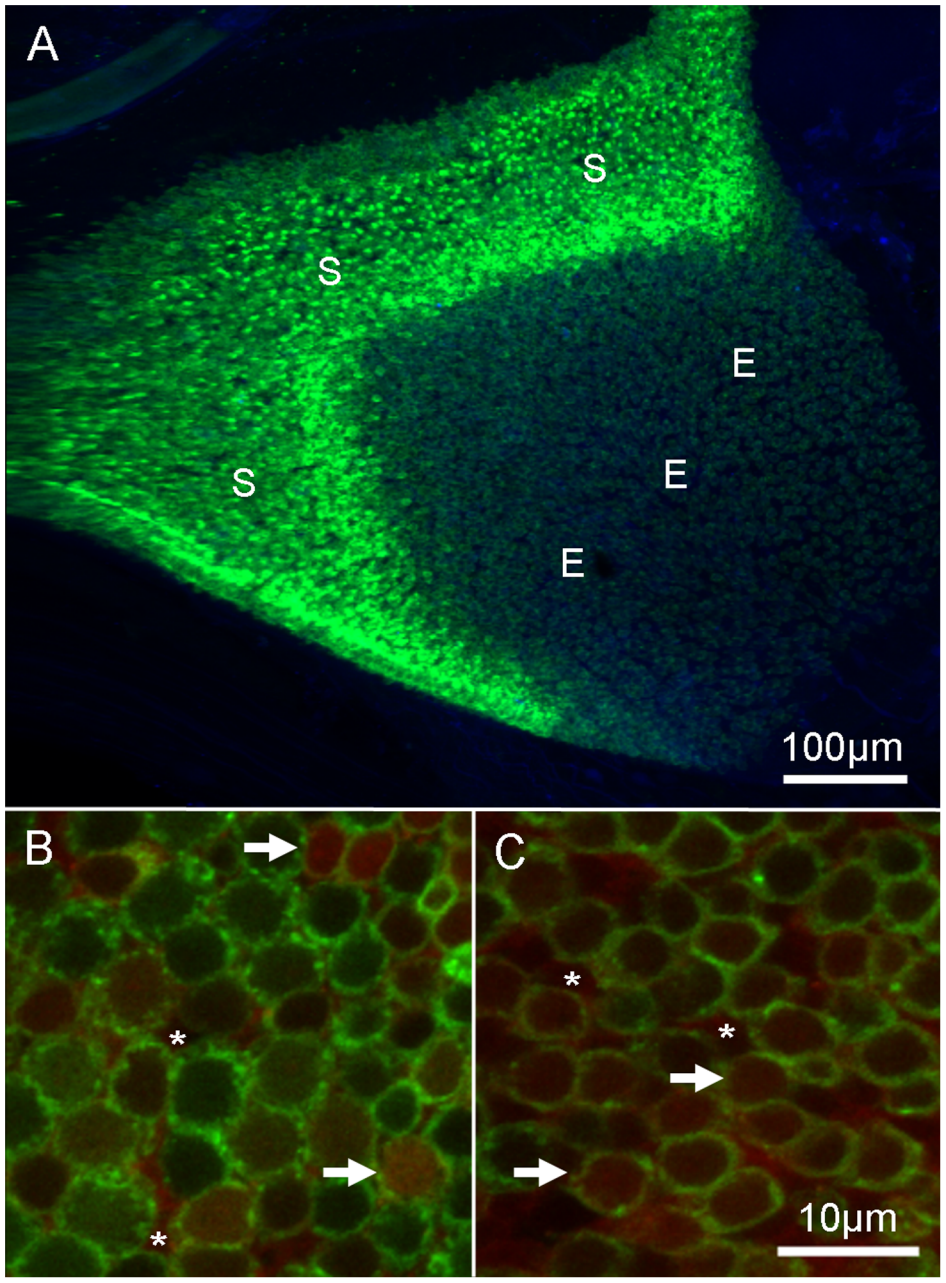
Gentamicin localizes to utricular hair cells. Adult Brn3c-GFP zebrafish received an intraperitoneal injection of GTTR (30 mg/kg) and allowed to recover for 4 hours. (A) Low magnification image of the utricle. GFP-labeled hair cells (green) are located throughout the organ. The striolar region (S) and extrastriolar region (E) are highlighted. High magnification images (B, C) are single optical slices from individual *z*-stacks from (B) striolar and (C) extrastriolar regions. GTTR fluorescence (red) was localized to hair cells (white arrows) and supporting cells (white asterisks) throughout the sensory epitheilum. GFP fluorescence (green) is localized to the cell membrane region of hair cells. The slices are taken mid-way through the cell to show the localization patterns, thus, neither hair cell nuclei nor stereociliary bundles are visible.

To determine cellular localization of GTTR, isolated organs were permeabilized with 0.1% Triton X-100 (Sigma) in PBS (PBST) for 30 minutes and then incubated with the cyanine monomeric dye TO-PRO-3 (1∶1000 in PBST; Life Technologies) for 2–3 hours. TO-PRO-3 binds to DNA and fluorescently labels nuclei. The organs were rinsed with PBS, mounted onto glass slides in Vectashield (Vector Labs, Burlingame, CA), and cover-slipped. Four designated regions of the saccule (5%, 25%, 50%, and 75% along the rostral-caudal length of the organ; Fig. 1), or the extrastriolar and striolar regions of the utricle (Fig. 2), were imaged [27], [28]. Images were acquired using a Nikon C1 confocal microscope (Nikon Instruments Inc., Melville, NY). Single images and *z*-series stacks were manipulated with EZ-C1 software (Nikon Instruments).

### Auditory Evoked Potentials (AEP)

Twelve zebrafish ranging from 2.9–3.9 cm standard length and 303–731 mg wet mass were used (6 for control; 6 for gentamicin treatment) to measure the effect of systemic gentamicin administration on AEP’s. AEP is a non-invasive method of measuring neural responses to auditory stimuli in fish and other vertebrates [36], [37]. Each fish was anaesthetized with MS-222, restrained in a mesh sling, and suspended under water in a 19-L plastic vessel. Each fish was suspended so that the top of the head was approximately 6 cm below the surface of the water and 22 cm above the underwater speaker.

Stainless steel subdermal electrodes (27 gauge; Rochester Electro-Medical, Inc., Tampa, FL) were used to record AEPs. A reference electrode was inserted approximately 2 mm subdermally into the medial dorsal surface of the head between the anterior portion of the eyes while a recording electrode was placed 2 mm into the dorsal midline surface of the fish approximately halfway between the anterior insertion of the dorsal fin and the posterior edge of the operculae, directly over the brainstem. A ground electrode was placed in the tail musculature of the fish.

Sound stimuli were presented and AEP waveforms collected using SigGen and BioSig software running on a TDT physiology apparatus (Tucker-Davis Technologies Inc., Alachua, FL). Sounds were computer generated via TDT software and passed through a P1000 power amplifier (Hafler, Tempe, AZ) connected to a University Sound UW-30 underwater speaker (Electro-Voice, Burnsville, MN). Tone bursts were 15, 10, and 5 ms in total duration for 0.1 and 0.25, 4 and 6, and 0.8–4 kHz tones, respectively. Each tone pip had a 2 ms rise and fall time and were gated through a Hanning window similar to the conditions of other AEP studies [38], [39], [40]. Responses to each tone burst at each SPL were collected using the BioSig software package, with 400 responses averaged for each presentation. Auditory thresholds were determined at 10 frequencies for each fish (0.1, 0.25, 0.4, 0.6, 0.8, 1, 1.5, 2, 3, and 4 kHz). The SPLs of each presented frequency were confirmed using a calibrated underwater hydrophone (calibration sensitivity of –195 dB re 1 V/µPa; ±3 dB, 0.02–10 kHz, omnidirectional, GRAS Type 10CT, Denmark), placed in the same location where fish were held during AEP recording. Auditory thresholds were determined by visual inspection of AEP as in previous studies [38], [39]. Separate one-way ANOVAs were calculated for each frequency (i.e., 100 Hz control vs. 100 Hz gentamicin-treated fish) using MS Excel (Microsoft, Redmond, WA).

### TUNEL Labeling

To assess relative levels of cell death, isolated organs were processed for Terminal dUTP Nick-End-Labeling (TUNEL) using a previously published protocol [41] to label cells with DNA fragmentation indicative of apoptotic cells. Fish received either an injection of buffer or gentamicin and were allowed to recover for 4 or 24 hours. Fixed organs were permeabilized with proteinase K (10 µg/mL) for 40 minutes at room temperature. The samples were then refixed in 4% paraformaldehyde for 20 minutes and then rinsed with PBS three times for 10 minutes each. The saccules and utricles were processed using an ApopTag Fluorescein *In Situ* Apoptosis Detection kit (Chemicon/EMD Millipore, Billerica, MA) according to manufacturer directions. For each whole-mounted endorgan, images were taken with a Zeiss Axioplan 2 (Carl Zeiss Microscopy, Germany) epifluorescence microscope at 10X and 100X objective magnification and all TUNEL-labeled cells were counted manually. One-way ANOVAs (SYSTAT 13; Systat Software Inc., Chicago, IL) were used to test for differences in numbers of TUNEL-labeled cells between gentamicin-treated animals and buffer-injected controls for each labeled endorgan.

### Hair Cell Stereociliary Bundle Counts and Analysis

Fixed, excised saccules and utricles were rinsed with PBS three times for 10 minutes each at room temperature. Isolated organs were permeabilized with 0.1% PBST for 30 minutes. The stereociliary bundles were labeled with Alexa Fluor 488-conjugated phalloidin (1∶100; Life Technologies) in the dark for two hours at room temperature to visualize filamentous actin (F-actin) that are abundant in stereociliary bundles and cuticular plates. After incubation, endorgans were placed on glass slides mounted with Prolong Gold antifade reagent with 4′,6-diamidino-2-phenylindole (DAPI; Life Technologies) to label nuclei and then cover-slipped.

Low (20X objective) and high (100X objective) power images of the saccule were viewed under the FITC filter of a Zeiss Axioplan 2 epifluorescence microscope and were imaged using an AxioCam MRm camera, with image contrast adjusted for easy quantification of phalloidin-labeled hair cell stereociliary bundles. Five designated regions of the saccule (5%, 25%, 50%, 75%, and 95% along the rostral-caudal length of the organ) were imaged [27], [28] to determine rostral-caudal shifts in saccular hair cell bundle density, as used previously [23], [40]. These locations were chosen as they represent a wide range in hair cell densities [27] and sensitivity to varying frequencies along the rostral-caudal axis of the zebrafish saccule [42]. At each location, images were cropped to a 30 µm×30 µm box using ImageJ (National Institutes of Health, Bethesda, MD) and cell counts performed.

Whole-mounts of utricles were visualized on a Nikon Eclipse Ni Fluorescence Microscope (Nikon Instruments) using a 60X objective and video images were obtained using a Nikon DS-Qi1 Cooled CCD camera and NIS Elements software. Cell counts were made using NIS Elements by placing a 5,000 µm^2^ box on the screen and using the cell counter feature. Phalloidin-labeled cells were counted from three boxes of the extrastriolar region and three boxes distributed along the striolar region of each utricle. Care was taken to avoid the lateral regions of sensory epithelia that contain immature hair cells [43], [44], [45], [46] that are more resistant to aminoglycoside toxicity [9] or areas of potential dissection damage. Cell counts from the three boxes were averaged to obtain an estimate of the number of surviving hair bundles/5,000 µm^2^ for the striolar and extrastriolar regions of each specimen.

Quantitative data were subjected to either a two-tailed t-test assuming unequal variance (MS Excel) or One-way ANOVA (VassarStats, Vassar College, Poughkeepsie, NY). Post-hoc comparisons, when appropriate, used the Tukey-HSD test.

## Results

### Localization of Gentamicin in the Inner Ear

Gentamicin kills inner ear sensory hair cells in a variety of adult fish [47], [48], [49], [50], [51], yet no studies have reported its effects on sensory hair cells in the inner ear of adult zebrafish. In order to determine if gentamicin can reach the inner ear organs, adult Brn3c-GFP transgenic zebrafish were given a single intraperitoneal injection of Texas Red-conjugated gentamicin (GTTR) and allowed to recover for 4 hours. These zebrafish express green fluorescent protein (GFP) in the cell membranes of sensory hair cells [20], [30], [31]. The zebrafish were then sacrificed, the head fixed and the inner ear organs were dissected, mounted, and imaged. We focused on the saccule since it is thought to be the putative “auditory” organ in the zebrafish [21], [22], [23], [24]. Four designated regions of the saccule (5%, 25%, 50%, and 75% along the rostral-caudal length of the organ) were imaged (Fig. 1A) [27], [28] since they represent a wide range in hair cell density [27] and sensitivity to frequency [42]. We found GTTR fluorescence localized in GFP-labeled hair cells and supporting cells in all regions of the saccule (Fig 1B–E; n = 6 animals). We also found GTTR-labeled hair cells and supporting cells in the utricle (Fig. 2A) in both striolar (Fig. 2B) and extra-striolar regions (Fig. 2C). As a control experiment, zebrafish injected with unconjugated Texas Red, and examined in the same manner, had negligible levels of Texas Red fluorescence in saccular or utricular hair cells (n = 8; data not shown).

### Gentamicin Affects Zebrafish Swimming Behaviors

In order to assess hair cell death following gentamicin treatment, adult zebrafish received a single 250 mg/kg intraperitoneal injection of gentamicin before being placed into individual tanks to recover for 4 or 24 hours. A preliminary study using transgenic Brn3c-GFP zebrafish showed that a single 250 mg/kg injection of gentamicin resulted in a 50% decrease in hair cells in specific regions of the saccule, and lower concentrations were less toxic (data not shown). Roughly 25% of the gentamicin-injected animals that received 250 mg/kg experienced systemic toxicity and typically died within 30 minutes of injection. Of the fish surviving the first 30 minute post-injection period less than 5% died before being euthanized at the 4 hour or 24 hour time points. All surviving fish were euthanized at either the 4 or 24-hour time point and long-term health effects of acute gentamicin injection were not assessed. Additionally, preliminary trials of serial injections, given once daily, either did not show significant toxicity at lower gentamicin concentrations or resulted in a fatality rate over 50% at higher doses (>50 mg/kg). In those that died there was a noticeable loss of vestibular function with many fish swimming upside down or lying prone on their side prior to death. Gill movement was also sporadic indicative of systemic changes in respiration. Doses >250 mg/kg were fatal to over 50% of injected animals. Therefore, we chose to use 250 mg/kg to induce ototoxic lesions, with lower levels of mortality. Zebrafish that survived treatment had some vestibular deficits. Within minutes after injection, fish were observed swimming off of level horizontal axis with a positive pitch that was maintained until the end of the experiment.

### Effects of Gentamicin on Auditory Thresholds

**Figure 3:**
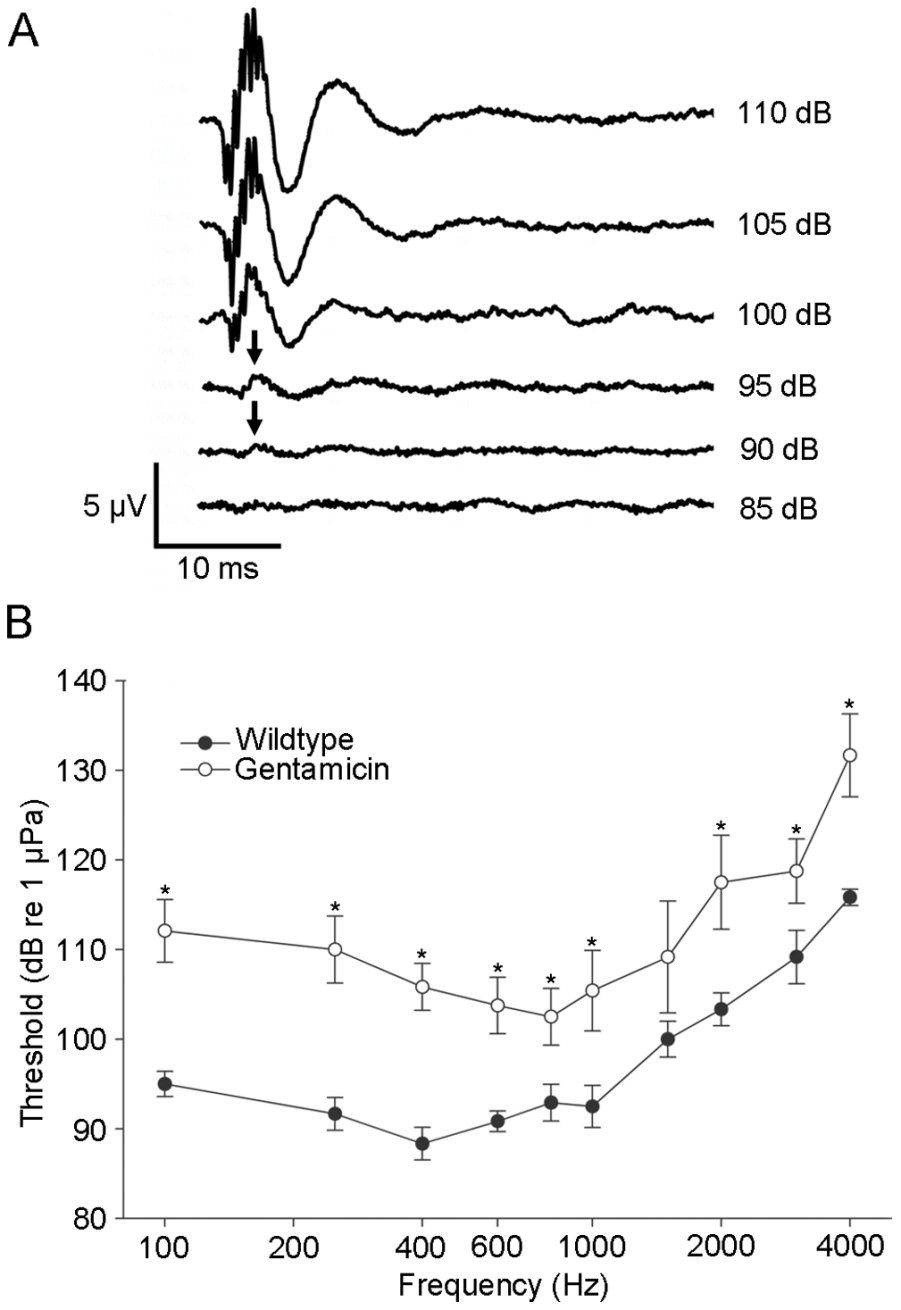
Gentamicin treatment causes auditory threshold shifts following 24 hours of treatment. (A) Electrophysiological recordings of auditory evoked potentials (AEPs) measured at a test frequency of 1000 Hz. Stimulus intensity was decreased from 110 dB to 85 dB in 5 dB steps. The AEPs emerge as a series of peaks (black arrows). In this example, the auditory threshold, as judged by visual inspection, is 90 dB (re: 1 µPa). (B) Auditory thresholds were used to construct audiograms to compare auditory function in treated and control fish. There was a significant auditory threshold shift (*p<0.05) at almost every frequency tested in gentamicin-treated fish when compared to untreated controls. n = 6 animals per treatment.

Auditory evoked potentials (AEPs) were recorded from untreated (wildtype) and gentamicin-treated animals. When we tried to record AEPs using the Brn3c-GFP transgenic zebrafish, the untreated control transgenic zebrafish had significant auditory threshold shifts compared to wildtype zebrafish (data not shown); therefore we used wildtype zebrafish for the remaining experiments. AEPs acquired from wildtype animals had stereotypic waveforms that emerged within 10 ms of the onset of the stimulus (Fig. 3A) similar to previous data for adult zebrafish [23], [24]. Audiograms were constructed by identifying the auditory thresholds (black arrow; Fig. 3A) in the gentamicin-treated and untreated control animals (Fig. 3B). There was a significant (p<0.05) threshold shift in wildtype zebrafish treated with gentamicin at every frequency tested except for 1500 Hz (n = 6 animals for each treatment group) 24 hours post-injection. This is likely due to the higher variability in AEPs from gentamicin-treated animals at this frequency.

### Cytotoxicity Analyses

**Figure 4:**
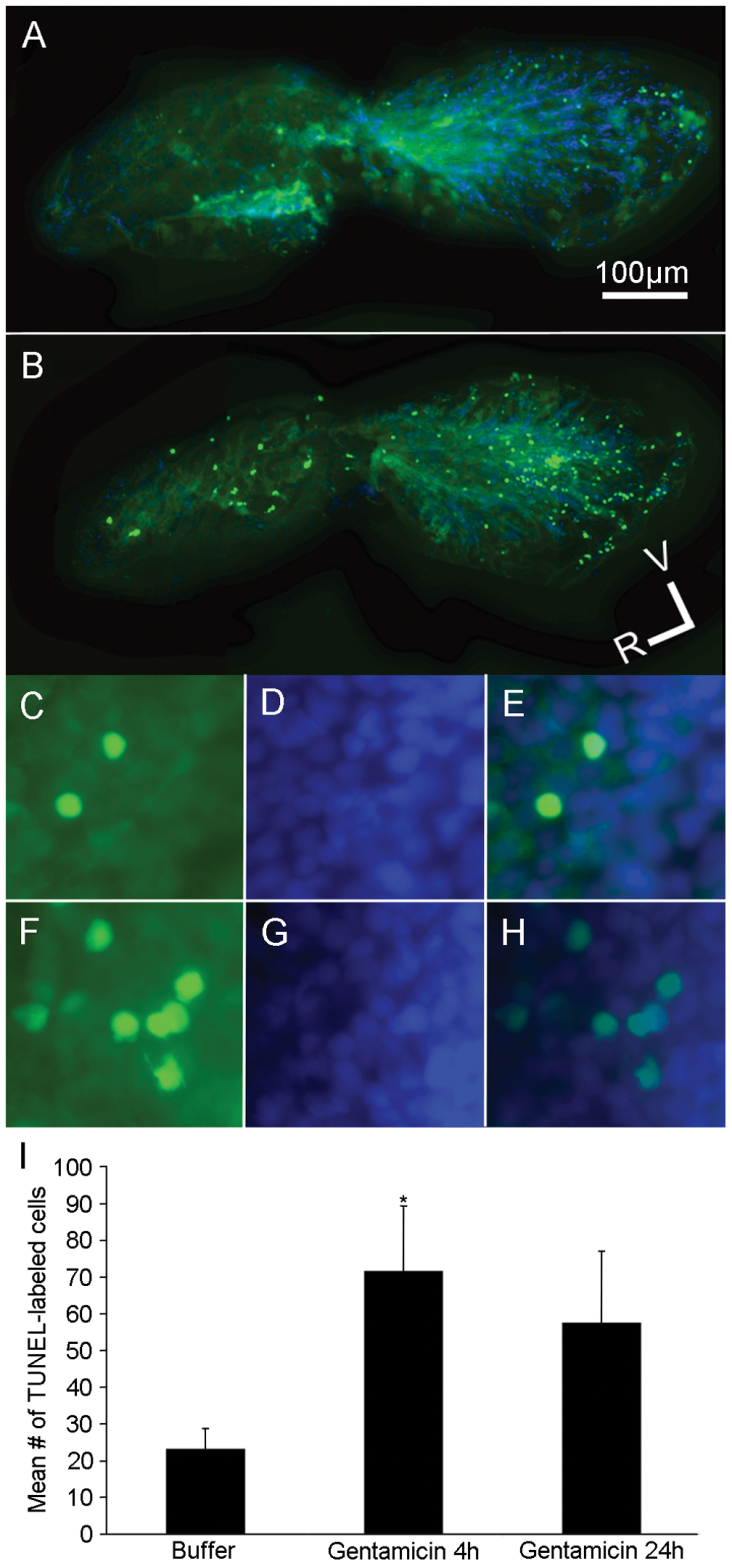
Gentamicin treatment increases the numbers of TUNEL-labeled cells in the adult saccule. Adult wildtype zebrafish received either a single intraperitoneal injection of (A, C, D, E) buffer or (B, F, G, H) 250 mg/kg gentamicin and allowed to recover for either 4 or 24 hours (4 hour data is shown). Fixed saccules were processed using a TUNEL-kit to label apoptotic cells (green cells; A, B, C, E, F, H) and co-labeled with DAPI (blue cells; A, B, D, E, G, H). (A-B) Low power and (C–H) high power images of the saccule reveals few TUNEL-labeled cells scattered throughout the saccule of a buffer-treated animal (A, C, E) but more TUNEL-labeling in a gentamicin-treated animal (B, F, H). (I) Mean number of TUNEL-labeled cells (± SD) in the saccules of buffer- or gentamicin-injected zebrafish. *p<0.05; n = 9–12; V = ventral, R = rostral.

**Figure 5:**
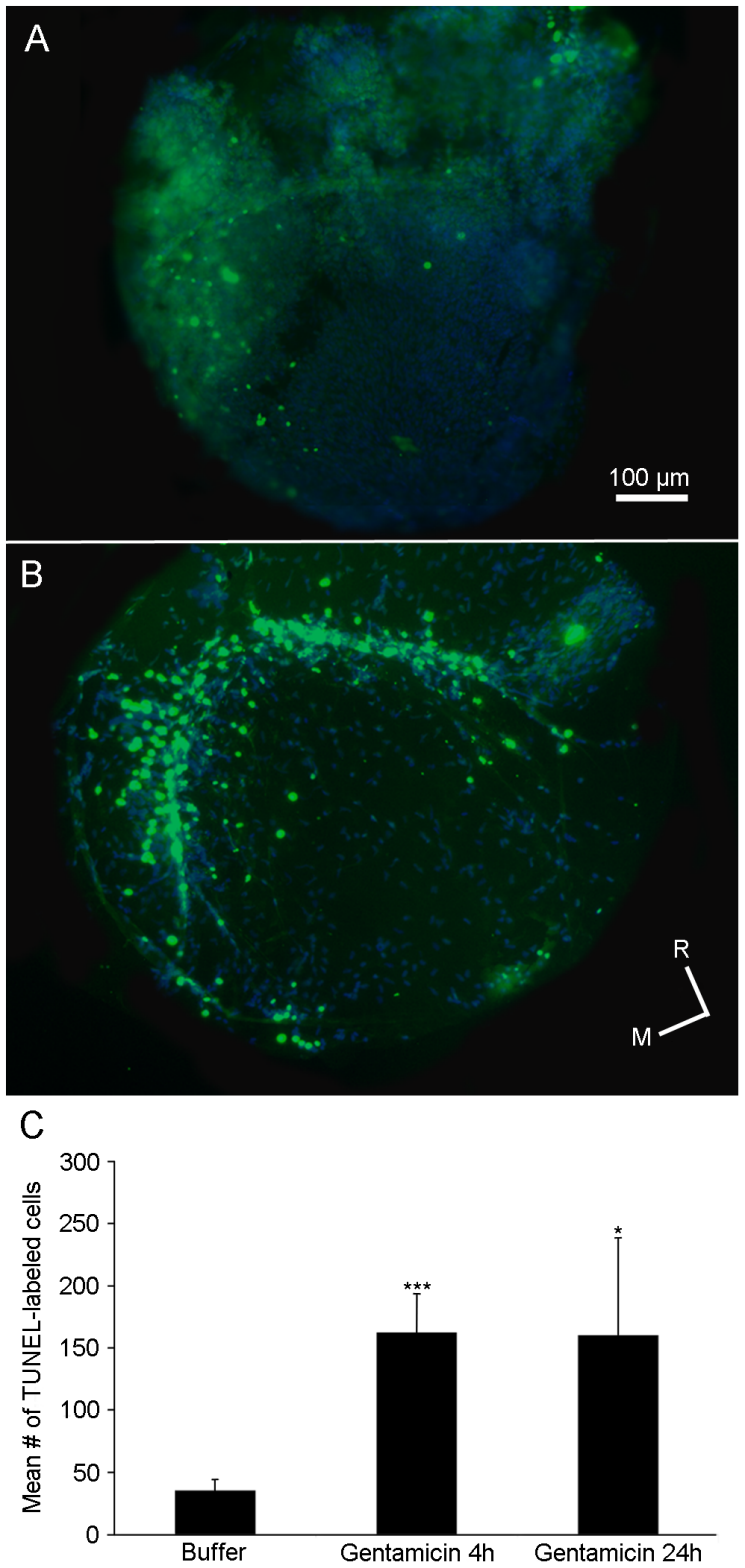
Gentamicin treatment increases the numbers of TUNEL-labeled cells in the adult utricle. Adult wildtype zebrafish received either a single intraperitoneal injection of (A) buffer or (B) 250 mg/kg gentamicin and allowed to recover for either 4 or 24 hours (4 hour data is shown). Fixed utricles were processed using a TUNEL-kit to label apoptotic cells (green cells) and co-labeled with DAPI (blue cells). (A–B) Low power (10X) images of the utricle reveals few TUNEL-labeled cells scattered throughout the utricle of a (A) buffer-treated animal but more cells with TUNEL-labeling in the (B) gentamicin-treated animal. (C) Mean number of TUNEL-labeled cells (± SD) in the utricles of buffer- or gentamicin-injected zebrafish. *p<0.05 or ***p<0.001; n = 9–12; M = medial, R = rostral.

In order to determine whether sensory hair cells died following gentamicin treatment, wildtype fish injected with gentamicin were allowed to recover for 4 or 24 hours. Some fish were injected with buffer and allowed to recover for 4 hours and served as controls. At the specified time, fish were fixed and processed for TUNEL labeling. Apoptotic cells can be detected by enzymatically labeling (TUNEL-labeling) the free 3′-OH termini of fragmented DNA with modified nucleotides (green cells; Figures 4 and 5). There was a significant increase in the number of TUNEL-labeled saccular (Fig. 4I; n = 9–12; p<0.05) and utricular cells (Fig. 5C; n = 12; p<0.001) 4 hours after gentamicin was administered compared to buffer-injected controls; this increase in the number of TUNEL-labeled cells was maintained 24 hours after gentamicin injection in the utricle (Fig. 5C; n = 9; p<0.05) although this was not statistically different from controls in the saccule (Fig. 4I; n = 9; p = 0.11).

**Figure 6:**
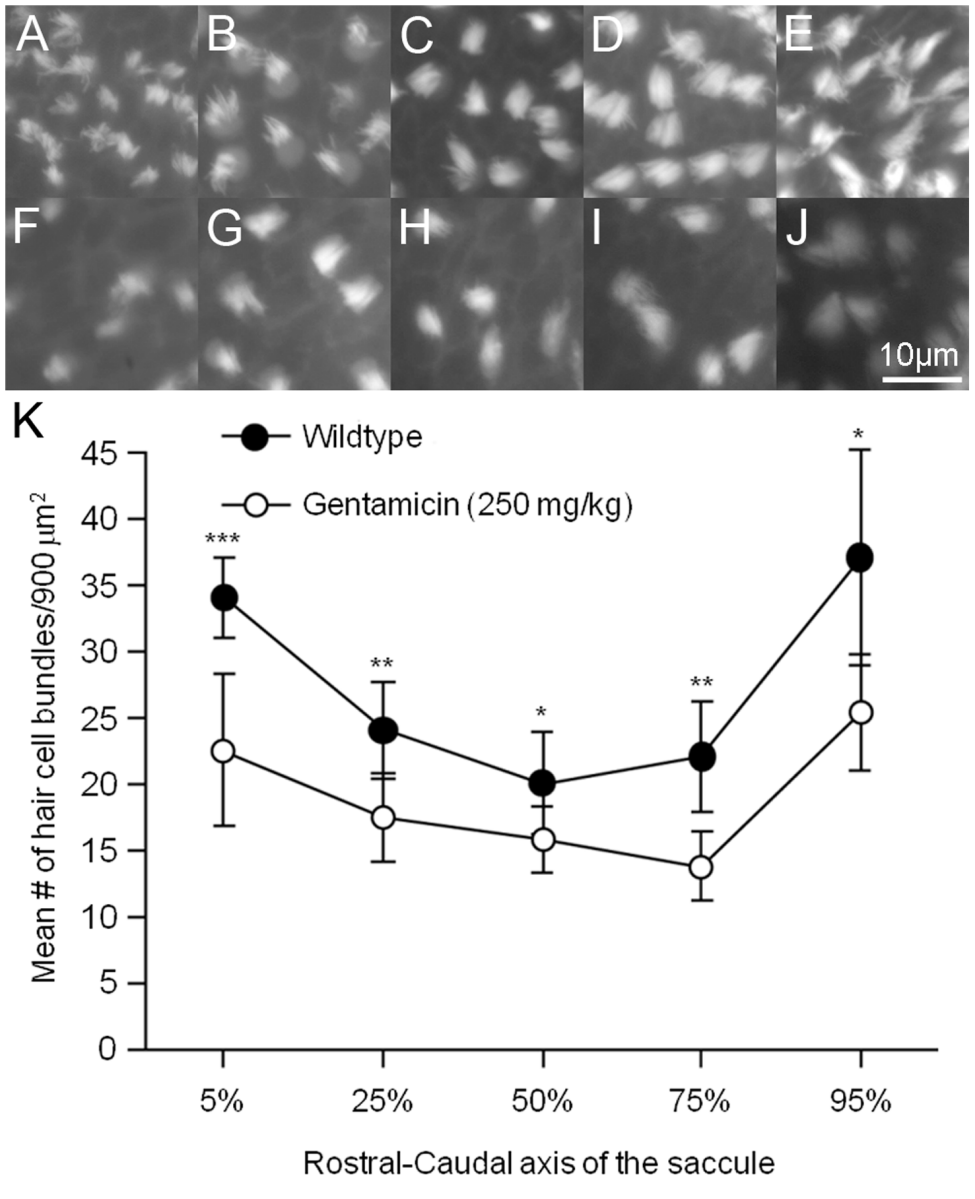
Gentamicin treatment resulted in fewer saccular hair bundles after 24 hours. Adult wildtype zebrafish received a single 250 mg/kg intraperitoneal injection of gentamicin and allowed to recover for 24 hours. Images of phalloidin-labeled cells were taken at (A, F) 5%, (B, G) 25%, (C, H) 50%, (D, I) 75%, and (E, J) 95% along the length of the saccule beginning at the rostral end in (A–E) untreated controls and (F–J) gentamicin-treated animals. (K) Mean (± SD) number of saccular hair cell bundles per 900 µm^2^ of epithelia. Significantly fewer phalloidin-labeled hair bundles were counted at each area along the length of the saccule in gentamicin-treated animals compared to controls. *p<0.05 or **p<0.01; n = 6–13 saccules per area per condition.

In order to determine if gentamicin treatment resulted in fewer sensory hair cells, actin filaments in the saccular and utricular hair cell stereociliary bundles in control and gentamicin-treated animals were specifically labeled with fluorescently-tagged phalloidin. There was a noticeable difference in the number of stereociliary bundles 24 hours after injection with 250 mg/kg gentamicin (Fig. 6F–J) compared to untreated controls (Fig. 6A–E). Hair bundle densities were significantly lower (p<0.05 to p<0.001) in gentamicin-treated saccules in every area sampled across the rostral-caudal axis (Fig. 6K, n = 6–12 per sample area per treatment group) 24 hours after of treatment.

Fewer sensory hair bundles were also identified in the utricle after gentamicin treatment. Phalloidin-labeled bundles in both the striolar and extrastriolar regions were quantified and expressed as the mean number of stereocilia bundles/5000 µm^2^± SD. Phalloidin-labeled bundles were counted from three regions in the central extrastriolar zone of each utricle and three regions throughout the striola (Fig. 2A). Hair bundle densities were greater in the striola (63.6±6.4; n = 7) than in extrastriolar regions (43.0±7.3; n = 7) of untreated control zebrafish. Four hours after 250 mg/kg gentamicin injection, there was a significant decrease in the number of stereociliary bundles (p<0.001) in both striolar region (49.1±10.1; n = 7) and the extrastriolar region (33.3±7.5; n = 8) compared to untreated controls. Twenty-four hours after the administration of gentamicin, there was still a significant decrease (p<0.001) in the number of stereociliary bundles in both striolar (51.9±6.5; n = 10) and extrastriolar regions (28.6±7.2; n = 10) compared to controls. While there were fewer stereociliary bundles in the extrastriolar region after 24 hours of treatment, there was no statistical difference between the two different time points (p>0.5).

## Discussion

Gentamicin-induced hair cell loss in the inner ear has been previously studied in several species of fish including oscar (*Astronotus ocellatus*) [47], [48], goldfish (*Carassius auratus*) [51], [52] and Atlantic Cod (*Gadus morhua*) [49], [50]. Different species respond to aminoglycosides differently. For instance, gentamicin kills hair cells in the lagena and utricle but not the saccule of the oscar [47]. In fact, no damage was reported in the saccule following higher doses of gentamicin [47]. In contrast, hair cells in the saccule of the goldfish [51] and Atlantic cod [49], [50], along with hair cells in both the utricle and saccule of the zebrafish, as reported here, are killed by systemic injection of gentamicin. We chose not to study the lagena since it is often damaged during excision of the saccule.

Hair cell uptake of GTTR in these vestibular organs (utricle and saccule) is variable, and this may underlie the susceptibility of some hair cells to undergo cytotoxicity and cell death compared to surviving hair cells in these organs. Modulation of gentamicin uptake by extracellular cations also modulates the susceptibility of lateral line neuromasts to cytotoxicity and cell death [15]. Variation in the uptake of aminoglycosides by inner ear hair cells in other fish species may also account for the observed variation in saccular and utricular hair cell death in differing species. Other experiments to measure aminoglycoside uptake in teleost inner ears are needed to determine this. In addition, the varied experimental paradigms also make comparison of their results more difficult to interpret. In three studies, repeated intramuscular injections of gentamicin to the oscar, goldfish, and Atlantic cod resulted in differing levels of damage. The Atlantic cod received multiple injections of gentamicin ranging from 5–80 mg/kg [49] whereas the oscars received multiple injections between 10–120 mg/kg. Unfortunately, further comparison between the goldfish study to the other two studies cannot be made as the dose given to goldfish was not reported. One major side effect of using repeated intramuscular injections are kidney problems as well as other health disorders in oscars [47], [48], goldfish [53], and Atlantic cod [49].

Some studies used an intra-saccular injection of gentamicin, which caused hair cell death in Atlantic cod [49], [50] and goldfish [54] since there are fewer side effects including less nephrotoxicity and death associated with this type of drug delivery. However, this procedure requires X-ray imaging in larger fish to determine if the needle is correctly positioned in the inner ear [49], and is not practical for smaller fish like zebrafish.

In this study, a single intraperitoneal injection of GTTR revealed trafficking of the conjugated drug into the inner ear, and that gentamicin caused significant saccular lesions and consequential auditory threshold shifts. GTTR was present in both hair cells and supporting cells in the both the saccule and utricle. The presence of GTTR in supporting cells is unsurprising and has also been observed in supporting cells (and hair cells) of frog, mouse, and guinea pig sensory epithelia [33], [55], [56].

While the dose of gentamicin in the current study was greater than in other teleost studies, these fish received only a single injection. Previous fish studies have given daily injections of gentamicin with varying onset of damage. For instance, the maximal amount of damage to the inner ear organs of the goldfish occurred 4 days after the beginning of gentamicin injections [51] versus the damage we observed 4 hours after a single injection. The onset of damage in the goldfish was not noted [51]. Gentamicin treatment can result in high mortality rates in zebrafish. Yet, a single dose was shown to be sufficient to produce a decrease in the number of hair cells. Four hours after injection of gentamicin, a noticeable increase in the number of apoptotic cells (as assessed by TUNEL labeling) and significant decrease of sensory hair cells in both saccules and utricles were observed. This is similar to lateral line neuromast hair cells. Within 4 hours of exposure to gentamicin or another aminoglycoside, neomycin, there are significantly fewer neuromast hair cells in larval zebrafish [7], [17]. Other studies of larval zebrafish show that functional mechanotransduction channels are required for GTTR to enter neuromast hair cells [56].

There have been studies in other species that compared a single injection versus multiple injections of gentamicin. In chickens, a single high dose of gentamicin (100 mg/kg) resulted in hair cell loss in the basal 20% of the cochlea and this occurred several days after injection [57]. Alternatively, multiple injections of gentamicin (50 mg/kg) for ten days resulted in the loss of the basal hair cells with a corresponding high-frequency hearing loss as measured by auditory brainstem responses [58], [59]. Other studies have reported variation in susceptibility to systemic and audiological toxicity to different aminoglycosides among different mammals including guinea pigs, rats, humans, and different strains of mice [60], [61]. Mechanisms of ototoxicity are not yet sufficiently understood to explain variation across species.

The speed with which gentamicin can induce zebrafish inner ear hair cell death and hearing loss has experimental advantages for studying hair cell regeneration. Most studies examining hair cell regeneration in the teleost inner ear have used an acoustic stimulus (either white noise or a low-frequency tone) that is played continuously for 40–48 hours to induce hair cell death and hearing loss [27], [28], [40]. Although minimal hearing loss (approximately 5 dB) is evident in goldfish within 10 minutes of white noise exposure, maximal threshold shifts occur approximately 24 hours later [38], [39]. It has been assumed that it takes approximately one day for acoustically-damaged hair cells to become non-responsive, begin to undergo apoptosis, and begin to be ejected out of the sensory epithelia, although this has not been directly measured via hair cell counts or cell death assays at time points earlier than 40–48 hours post-sound exposure [27], [28], [40]. The gentamicin-injection protocol presented in this paper will be useful for future comparisons between acoustically- and ototoxic chemical-induced hair cell death and regeneration.

Previous studies using goldfish have shown that multiple intramuscular injections of gentamicin can cause auditory hair cell death and hearing loss [51], [52]. In these studies, hearing loss was limited to frequencies between 300 and 600 Hz, whereas in this zebrafish study, hearing loss was evident at almost all frequencies between 100 and 4000 Hz. The extent of hearing loss (approximately 8–15 dB), though, was similar where threshold shifts were evident in both sets of studies. It is likely that differences in the two studies are a result of gentamicin dosage, rather than species differences since goldfish and zebrafish are closely-related cyprinids with similarly-shaped audiograms and inner ear structure [62]. Unfortunately, the gentamicin dosage cannot be directly compared between the two studies since the goldfish studies reported injected dosages as mg/ml without specifying the total volume injected per fish.

This study demonstrated the ototoxic effects of gentamicin on the saccule and utricle of adult zebrafish. Future studies will evaluate the effects of other aminoglycosides and the chemotherapeutic agent cisplatin on the inner ear hair cells of adult zebrafish. In addition, subsequent studies will evaluate the regeneration of inner ear sensory hair cells and auditory evoked potentials after gentamicin treatment and compare this recovery to studies that have investigated acoustically-induced damage in adult zebrafish [27], [28], [29].
